# New molecular sequences for two genera of marine planarians facilitate determination of their position in the phylogenetic tree, with new records for two species (Platyhelminthes, Tricladida, Maricola)

**DOI:** 10.3897/zookeys.781.26324

**Published:** 2018-08-09

**Authors:** Hee-Min Yang, Ronald Sluys, Masaharu Kawakatsu, Gi-Sik Min

**Affiliations:** 1 Department of Biological Sciences, Inha University, Incheon, Republic of Korea Inha University Incheon Korea, South; 2 Naturalis Biodiversity Center, P.O. Box 9517, 2300 RA Leiden, The Netherlands Naturalis Biodiversity Center Leiden Netherlands; 3 9-jo 9-chome 1-8, Shinkotoni, Kita-ku, Sapporo, Hokkaido, Japan Unaffiliated Sapporo Japan

**Keywords:** Antarctica, molecular phylogeny, new records, *
Obrimoposthia
*, *
Paucumara
*, South Korea

## Abstract

For the first time, molecular sequences of the 18S ribosomal DNA were generated for representatives of the genera *Obrimoposthia* Sluys & Ball, 1989 and *Paucumara* Sluys, 1989 of the suborder of the marine triclads, or Maricola, by analyzing the species *Obrimoposthiawandeli* (Hallez, 1906) and *Paucumaratrigonocephala* (Ijima & Kaburaki, 1916). On the basis of this molecular data the phylogenetic position of these two genera in the phylogenetic tree of the Maricola was determined and compared with their position in the phylogeny based on the analysis of anatomical features. New records for these two species are documented and their taxonomic status is determined on the basis of histological studies.

## Introduction

Although the marine planarians or Maricola Hallez, 1892 form only a small suborder of triclad flatworms, comprising approximately 80 species, they exhibit a rather great anatomical diversity, which at times makes it difficult to recognize homological character states and thus to analyse their phylogenetic relationships. The first comprehensive study of the phylogenetic relationships among the marine triclads was undertaken by Sluys (1989) and was based on anatomical and morphological features. As morphological support for a monophyletic Maricola was postulated the autapomorphic presence of adhesive papillae arranged in the ventral annular zone, the latter constituting an autapomorphy for the entire group of triclads (Sluys 1989). More recently, Ax (2008) correctly argued that this marginal band with adhesive papillae is a plesiomorphic feature and therefore cannot support the presumed monophyly of the Maricola. Nevertheless, recent phylogenetic studies on the triclads consistently recover the Maricola as a monophyletic taxon (see Charbagi-Barbirou et al. 2011, Sluys et al. 2014, Harrath et al. 2016). The same molecular studies reveal relationships within the Maricola that differ from those hypothesized by Sluys (1989), thus suggesting that eventually major changes in the current taxonomy of the group may be necessary.

Unfortunately, the number of species incorporated in molecular phylogenetic studies of marine planarians is rather small, as for only a handful of species gene sequences are publicly available. Therefore, it is as yet not possible to draw firm conclusions about the phylogeny, and thus the taxonomy, of maricolans based on such molecular studies. In this paper we contribute to the solution of this problem by making available gene sequences of two other species of marine planarian, *Paucumaratrigonocephala* (Ijima & Kaburaki, 1916) and *Obrimoposthiawandeli* (Hallez, 1906), which previously have not been examined. On the basis of this molecular data we analyze the phylogenetic position of the respective genera in the phylogenetic tree of the Maricola and compare this result with their position in the phylogeny based on the analysis of anatomical features. In addition, we document new records for these two species as well as their taxonomic status, as deduced from histological studies.

## Materials and methods

Collected worms were transferred live to the laboratory, where specimens of *Paucumaratrigonocephala* and *Obrimoposthiawandeli* were incubated under dark conditions at temperatures of 18 °C and 4 °C, respectively. For morphological study specimens were killein 10% glacial acetic acid and, subsequently, fixed for 24 hour in Bouin’s fluid, and stored in 70% ethanol. For histological processing specimens were first dehydrated in a graded ethanol series, cleared in clove oil and then embedded in synthetic wax. Serial sections were made at intervals of 5 μm and 8 μm, mounted on albumen-coated slides, stained with Mallory-Heidenhain/Cason (see Winsor and Sluys 2018), and, subsequently, cover glasses were mounted with DPX. Reconstructions of the copulatory complex were obtained by using a camera lucida attached to a compound microscope. The material is deposited in the National Institute of Biological Resources (NIBR), Seoul, Republic of Korea and Naturalis Biodiversity Center, Leiden, The Netherlands (ZMA collection code).

Before performing the molecular analysis, specimens of *P.trigonocephala* and *O.wandeli* were first starved for more than seven days. Genomic DNA was extracted, using LaboPass^M^ Tissue Mini Kit (Cosmogenetech, Seoul, South Korea), from either starved live worms or from 100% ethanol fixed specimens that before fixation had been starved also for seven days. We obtained two 18S ribosomal DNA sequences from both species. To infer their position in the phylogenetic tree of the triclads we constructed Bayesian Inference (BI) and Maximum-likelihood (ML) trees, using 24 planarians as ingroup and three fecampiid species as outgroup taxa (Table 1).

**Table 1.** List of species taxa of which 18S rDNA gene data was used for constructing the phylogenetic trees, with accession numbers for gene sequences available from GenBank.

Classification	Species	GenBank number
Suborder Maricola Hallez, 1892		
Family Cercyridae Böhmig, 1906	* Cercyra hastata *	KM200902
	* Sabussowia dioica *	JN009785
	* Sabussowia ronaldi *	KM200923
Family Uteriporidae Böhmig, 1906	* Ectoplana limuli *	D85088
	* Obrimoposthia wandeli *	MH108586
	* Paucumara trigonocephala *	MH108587
	* Sluysia triapertura *	MF383119
	*Uteriporus* sp.	AF013148
Family Bdellouridae Diesing, 1862	* Bdelloura candida *	Z99947
	* Palombiella stephensoni *	DQ666008
	* Pentacoelum kazukolinda *	KM200905
Family Procerodidae Diesing, 1862	* Procerodes dohrni *	JN009783
	* Procerodes littoralis *	Z99950
	* Procerodes plebeius *	DQ665997
Incertae sedis	*Maricola* sp.	KC869825
Suborder Cavernicola Sluys, 1990		
	*Cavernicola* sp.	KC869823
	* Novomitchellia bursaelongata *	KU096054
Suborder Continenticola Carranza, Littlewood, Clough, Ruiz-Trillo, Baguñà & Riutort, 1998		
Family Dugesiidae Ball, 1974	* Dugesia gonocephala *	DQ666002
	* Dugesia japonica *	D83382
	* Dugesia ryukyuensis *	AF050433
	* Dugesia subtentaculata *	AF013155
Family Planariidae Stimpson, 1857	* Crenobia alpina *	M58345
	* Phagocata vitta *	DQ665998
	* Polycelis felina *	DQ665996
Order Fecampiida Rohde, Luton & Johnson, 1994		
	* Kronborgia isopodicola *	AJ012513
	*Piscinquilinus* sp.	AJ012512
	* Urastoma cyprinae *	AF167422

The 18S ribosomal DNA gene was amplified using Polymerase Chain Reaction (PCR) with four primers: 1F, 4F, 7R, 9R (see Carranza et al. 1996). The PCR amplifications were conducted in a final volume of 35µL under the following conditions: 2 min at 94 °C, 40 cycles of 20 s at 95 °C, 30 s at 45 °C, and 1min at 72 °C, and 5min at 72 °C as a final extension. After purifying PCR products, using LaboPass^M^PCR Purification Kit (Cosmogenetech, Seoul, Republic of Korea), 18S gene sequences were determined from both strands by Macrogen Inc. (Seoul, Republic of Korea) by using the 3730xl DNA analyser with the same primers that were used in the PCR. For constructing the 18S rDNA phylogenetic tree, we used genomic data of 27 species, including three outgroup taxa (Table 1). The sequences were aligned using MAFFT version 7 (Katoh et al. 2017) (using the G-INS-i iterative refinement method and with the other options set as default) and checked using BioEdit 7.2.6.1 (Hall 1999). Regions that could not be clearly aligned were excluded using Gblocks version 0.91b (Castresana and Talavera 2007) (with the option of half allowed gap positions and minimum length of a block being 6). The final length of the alignment was 1,398bp. To find the best-fit evolutionary model, we used Jmodeltest2 (Darriba et al. 2012). GTR+I+G model was selected by applying the Akaike information criterion. We used Mr Bayes v 3.2 (Ronquist et al. 2012) and RaxML 8.2.10 (Stamatakis 2014) to infer phylogenies with the Bayesian Inference (BI) and Maximum-likelihood (ML) method, respectively. For BI method, two runs for 5 million generations and 25% burn-in was used under the GTR+I+G model. For the ML analysis, we performed 10,000 replicates with the same model. BI and ML trees were visualized using Figtree v1.4.3 and edited by using Adobe^R^ Photoshop^R^ CS5.

### Abbreviations used in the figures

**bc** bursal canal;

**br** brain;

**ca** common atrium;

**cb** copulatory bursa;

**cod** common oviduct;

**cvd** common vas deferens;

**ed** ejaculatory duct;

**el** eye lens;

**go** gonopore;

**in** intestine;

**mo** mouth opening;

**od** oviduct;

**pb** penis bulb;

**pg** penis gland;

**ph** pharynx;

**pp** penis papilla;

**sg** shell gland;

**spt** septum;

**ug** unicellular gland;

**vd** vas deferens;

**vi** vitellaria

## Results

### Phylogeny

**Figure 1.**
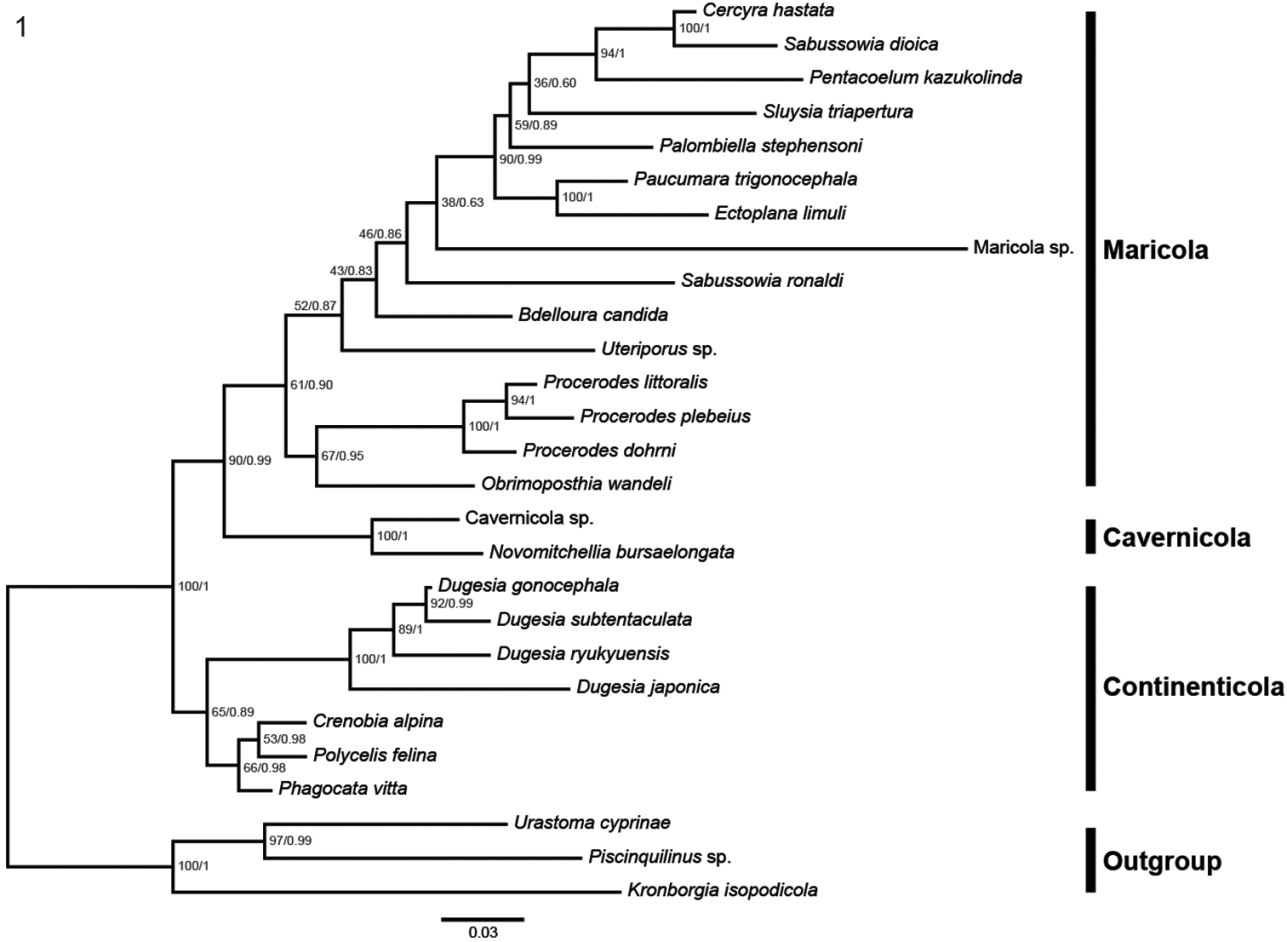
Maximum-likelihood tree based on 18S ribosomal DNA sequences. Numbers on nodes represent support values for Maximum-Likelihood (bootstrap –BO) and Bayesian Inference (posterior probability – PP): BO/PP. Scale bar indicates number of nucleotide substitutions per site.

The BI and ML phylogenetic trees showed the same topology (Figure 1). The suborders Cavernicola Sluys, 1990, Continenticola Carranza et al., 1998, and several of their inclusive lower taxa, form monophyletic groups. The Maricola Hallez, 1892 is also a monophyletic group, although the relationships within this group do not always reflect current taxonomy. For example, only the family Procerodidae Diesing, 1862 forms a monophylum, while current families Bdellouridae Diesing, 1862, Uteriporidae Böhmig, 1906 and Cercyridae Böhmig, 1906 form polyphyletic groups in our tree.

In the phylogenetic tree, the inferred positions of species belonging to the current families Bdellouridae, Uteriporidae and Cercyridae are supported only by BI, as the bootstrap supports for ML are < 75. The species *Cercyrahastata* Schmidt, 1861, *Sabussowiadioica* (Claparède, 1863), *Pentacoelumkazukolinda* (Kawakatsu & Mitchell, 1984), *Paucumaratrigonocephala* and *Ectoplanalimuli* (Ijima & Kaburaki, 1916) form an exception, in that their positions in our tree are supported by both ML and BI.

*Paucumaratrigonocephala* and *Obrimoposthiawandeli* are currently classified as belonging to the Uteriporidae. In our phylogenetic tree *P.trigonocephala* forms a highly supported clade with *Ectoplanalimuli*, currently also classified as an Uteriporidae species. The position of *O.wandeli* is also inferred with high posterior probability value in the BI tree and this species forms a clade with the family Procerodidae, here represented by three species included in our analysis. However, in the ML tree (not shown but with the same topology as the BI tree), the clade formed by the three species of *Procerodes* and *Obrimoposthia* (Figure 1) had only a bootstrap support value of 67.

The unidentified species Maricola sp. shows a very long branch that differs strongly from the branch lengths of other maricolans. Together with the low support values (bootstrap: 38; posterior probability: 0.63) this suggests that the molecular sequence of Maricola sp. may be corrupted.

### Systematic and integrative account

#### Order TRICLADIDA Lang, 1884

##### Suborder MARICOLA Hallez, 1892

###### Family UTERIPORIDAE Böhmig, 1906

####### Genus *Paucumara* Sluys, 1989

######## Paucumara trigonocephala

Taxon classificationAnimaliaTricladidaUteriporidae

(Ijima & Kaburaki, 1916)

######### Material examined.

NIBRIV0000821277, Sacheon-si, Gyeongsangnam-do, Republic of Korea (35°05'05"N 128°03'14"E), 7 June 2017, coll. H-M. Yang, sagittal sections on 2 slide; ZMA V.Pl. 7279.1, ibid., sagittal sections on 3 slides; V.Pl. 7279.2, ibid., horizontal sections on 1 slide; V.Pl. 7279.3, ibid., transverse sections on 6 slides.

ZMA V.Pl. 6807, shore of Lake Hi-numa, near the Park, Ibaraki-machi, Higashi-Ibaraki-gun, Ibaraki Prefecture, Kantô Region, Honshû, Japan, 13 August 2007, coll. S. Chinone, preserved specimens. ZMA V.Pl. 6810, ibid., 31 August 2007, coll. S. Chinone, preserved specimens.

######### Comparative description and discussion.

**Figures 2–3.**
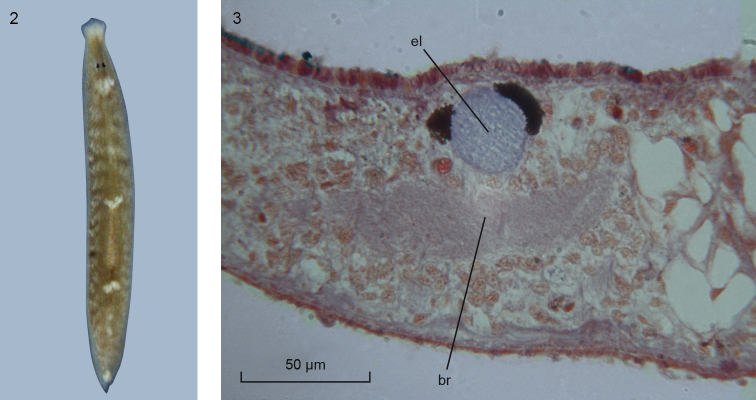
*Paucumaratrigonocephala*. **2** Dorsal view of live specimen from South Korea. Scale bar not available **3**ZMA V.Pl. 7279.1, microphotograph of eye lens; anterior to the left.

The external features and anatomy of the specimens from South Korea correspond in all essential details to the descriptions of this species published earlier (see Sluys 1989, Sluys and Ball 1990, Sluys and Kawakatsu 2000). Preserved specimens measured up to approx. 3 mm in length and 1 mm in width. In particular, the shape of the body and the position of the eyes in living specimens (Figure 2) are very similar to the situation in Australian specimens, as documented in Sluys (1989) and Sluys and Kawakatsu (2000). The two eyes are set close to the mid-line of the body and positioned at a considerable distance posterior to the anterior body margin.

The shape of the front end of the body is very characteristic: anterior to the eyes the body first narrows to give rise to a kind of “neck” and then widens to form a triangular, obtusely pointed head with broadly rounded auricles (Figure 2). At the level of the auricles there is a broad, creamy-white patch that extends across the body. A similar kind of patch is located immediately behind the eyes, albeit that it does not extend from one lateral body margin to the other but is confined to mid-dorsum. Additional creamy-white spots may be located directly in front of and behind the pharyngeal pocket and at the very tip of the tail. In point of fact, each of the pharyngeal patches may actually consist of two spots situated close together. These pharyngeal spots as well as the one at the tip of the tail were not present in every specimen examined and neither were they reported earlier in the available literature.

In the specimens from South Korea the entire dorsal surface is provided with a brownish pigmentation. The pigment granules are more or less evenly distributed, but accumulations occur in front of the eyes, where there is a broad, transverse band, and in the form of a brown stripe on either side of the pharyngeal pocket and a band of brown pigment running between the eyes. A brownish colouration, on both dorsal and ventral body surface, was described also for specimens from northern Australia (Sluys and Kawakatsu 2000).

**Figure 4.**
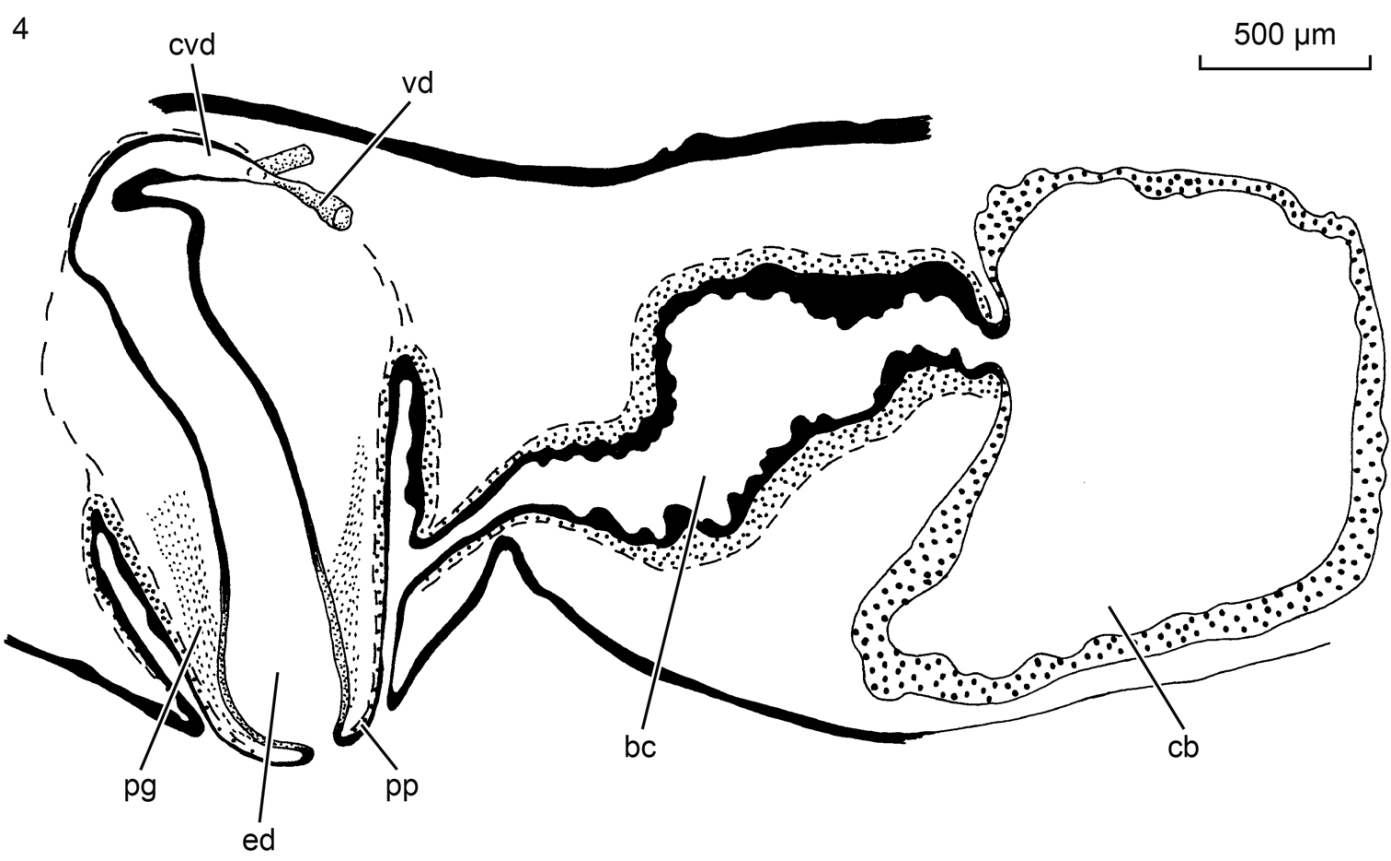
*Paucumaratrigonocephala*. ZMA V.Pl. 7279.1, sagittal reconstruction of the copulatory apparatus; anterior to left.

**Figures 5–6.**
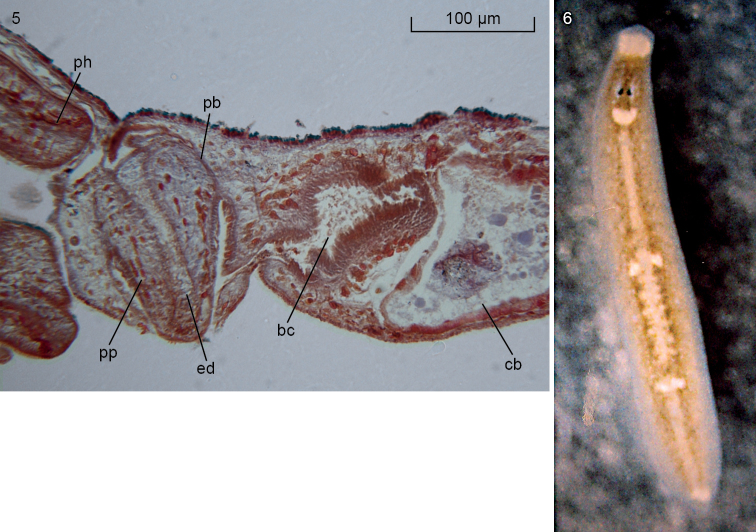
*Paucumaratrigonocephala*. **5**ZMA V.Pl. 7279.1, microphotograph of sagittal section of the copulatory apparatus; anterior to the left **6** Dorsal view of live specimen from Lake Hi-numa, Japan. Scale bar not available.

With respect to their anatomy, the South Korean animals exhibit a distinct lens in each of their eyes (Figure 3) and a copulatory apparatus (Figs 4, 5) similar to that documented for animals from other parts of the range of the species (see Sluys and Ball 1990, Sluys and Kawakatsu 2000, and references therein). The penis papilla is a stubby cone. Immediately after having penetrated the penis bulb, the vasa deferentia unite to form a common vas deferens, which communicates with a much broader ejaculatory duct. At its distal, ventral section the latter receives the openings of erythrophilic penis glands. The sac-shaped copulatory bursa fills the entire dorso-ventral space; it is connected with the common atrium by means of a bursal canal that is lined with an infranucleated epithelium. The major portion of the bursal canal is rather wide and irregularly shaped, but the part near its opening into the common atrium is narrow. This lower, proximal portion of the bursal canal receives the separate openings of the oviducts. Unfortunately, in specimens NIBRIV0000821277, ZMA V.Pl. 7279.1, and ZMA V.Pl. 7279.2 we were unable to trace the oviducts and only in the transversally sectioned specimen ZMA V.Pl. 7279.3 did we observe the oviducts separately opening into the bursal canal. The entire bursal canal is covered with a well-developed, subepithelial layer of circular muscle, followed by a thin layer of longitudinal muscle.

According to Sluys (1989), Sluys and Ball (1990), and Sluys and Kawakatsu (2000), the bursal canal receives along its entire length numerous openings of erythrophilic glands, the cell bodies of which are located ectally to the surrounding coat of muscle. These glands are different from the shell glands, which open into the bursal canal ectally to the oviducal openings. In the present material we observed that indeed at places a granular, erythrophilic secretion is discharged into the lumen of the bursal canal. However, this secretion is not easy to detect and apparently in our specimens this situation is much less developed than as described in the literature for other specimens of *Paucumaratrigonocephala*. We were unable to detect shell glands in our animals from South Korea.

Previous records of *Paucumaratrigonocephala* from Japan, Australia, the Bismarck Archipelago and probably Hong Kong were summarized in Sluys and Ball (1988), Sluys (1989), and Sluys and Kawakatsu (2000). We do here document a new locality for Japan, viz., Lake Hi-numa; this is a lake that is connected with the sea and, thus, has a low level of salinity. Histological sections have not been prepared of the preserved specimens available from this locality (see Material examined) but a picture of a live specimen (Figure 6) leaves little doubt about its specific identity. It is noteworthy that this animal also exhibits the whitish patches in the pharyngeal region and at the tip of the tail (see above).

Although the species was probably observed in Hongkong as early as 1857 (see Sluys 1989) our present animals from South Korea represent the first substantiated record of *P.trigonocephala* from continental Asia. Here, the animals were collected from brackish water with a low salinity level, the bottom consisting of mud with small stones and being devoid of any aquatic vegetation. This habitat is in agreement with the fact that all over its range the animals show the same characteristic ecology, in that they live in low-salinity biotopes, which during ebb tide even may be entirely fresh (Kaburaki 1922, Sluys and Kawakatsu 2000).

In the molecular phylogenetic trees generated by Souza et al. (2018) their new genus and species *Sluysiatriapertura* Leal-Zanchet & Souza, 2018 is the sister-group of the ectocommensal species *Ectoplanalimuli* (Ijima & Kaburaki, 1916), albeit that this relationship only has low support in their trees. In our tree (Figure 1) the topology has changed slightly in that *P.trigonocephala* has become the sister-species of *E.limuli*, while *S.triapertura* forms part of a group of five species that constitutes the sister-taxon of *E.limuli* plus *P.trigonocephala*. That *E.limuli* and *P.trigonocephala* share a sister-group relationship conforms with the current taxonomy of the Maricola, in which both species are classified amongst members of the subfamily Ectoplaninae Bresslau, 1933 (Sluys 1989).

### Genus *Obrimoposthia* Sluys & Ball, 1989

#### Obrimoposthia wandeli

Taxon classificationAnimaliaTricladidaUteriporidae

(Hallez, 1906)

synonym: Procerodessanderi [Hauser, 1987]

##### Material examined.

NIBRIV0000813547, King George Island, South Shetland Islands, 62°12'31"S – 58°47'42"W, 2 February 2017, coll. Hee-Min Yang, sagittal sections on 11 slides; ZMA V.Pl. 7280.1, ibid., sagittal sections on 14 slides. The animals were collected from a pebble beach; temperature of the sea surface water was 0~1°C; at a depth of 10m water temperature was 1~0°C. Many worms were observed to be attached to the seaweed Kelp.

ZMA V.Pl. 951.1, King George Island, South Shetland Islands, 1983, sagittal sections on 15 slides; V.Pl. 951.2, ibid., whole mount on 1 slide; V.Pl. 951.3, ibid., sagittal sections on 13 slides; V.Pl. 951.4, ibid., sagittal sections on 13 slides; V.Pl. 951.5, ibid., transverse sections on 19 slides; V.Pl. 951.6, ibid., horizontal sections on 8 slides.

Holotype *Procerodessanderi*: MZU PL. 00290, sagittal sections on 48 slides (nos. A-140/A-188).

MZU PL. 00291, sagittal sections on 30 slides (nos. A788-821) of presumed specimen of *Procerodessanderi* from the original collection of J. Hauser.

##### Comparative description.

Preserved specimens, collected in 2017, up to approx. 11 × 3.5 mm, thus being somewhat larger than reported for preserved animals of *Obrimoposthiawandeli* (Hallez, 1906), which measured 4–8 mm in length and 2.5–4 mm in width (Sluys 1989). However, much of this variation in size may be due to different fixatives used in the field, resulting in different states of contraction of the animals. Nevertheless, preserved specimens of the sample ZMA V.Pl. 951 also were quite large, measuring 8-10 × 3–5 mm.

**Figures 7–8.**
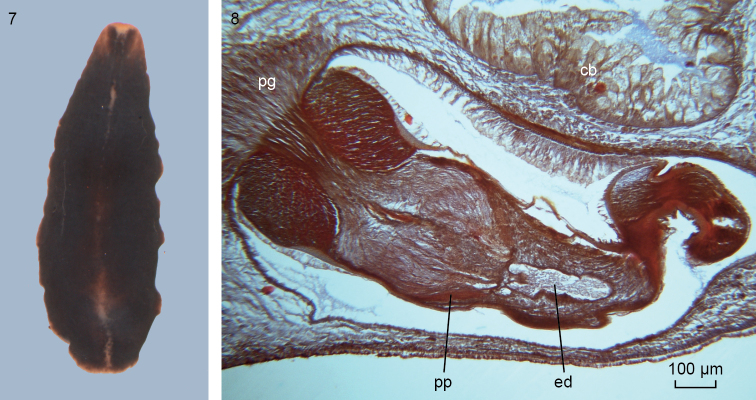
*Obrimoposthiawandeli*. **7** Dorsal view of preserved specimen from King George Island. Scale bar not available **8** NIBRIV0000813547, microphotograph of sagittal section of penis papilla; anterior to the left.

Dorsal surface of our animals from 2017 mottled blackish or dark brown, with a pale mid-dorsal stripe, which is only weakly developed on the middle portion of the body (Figure 7). At the front end of the body the dark pigmentation gives way to a broad, pale patch on either side of the body, extending from the eyes towards the antero-lateral body margins; further there is a pale patch on the mid-frontal body margin. In this way the dark pigmentation on the head forms a kind of V-shaped pattern; the same pattern was described for other specimens of *O.wandeli* (Sluys 1989). The external appearance and colouration of our 2017 specimens fully agree with that of specimens of *P.sanderi* as originally described in 1987 (see figure in Anonymous 1987).

In the specimens from the 2017 sample the small, rounded testes are situated ventrally and extend from immediately behind the ovaries to somewhat posteriorly to the copulatory apparatus, as may be the case also in other specimens of *O.wandeli* (Sluys 1989). The anatomy of the penis papilla of these specimens from the 2017 sample is precisely the same as that documented for *O.wandeli* (see Sluys and Ball 1988, Sluys and De Vries 1988, Sluys 1989) (Figure 8).

**Figure 9.**
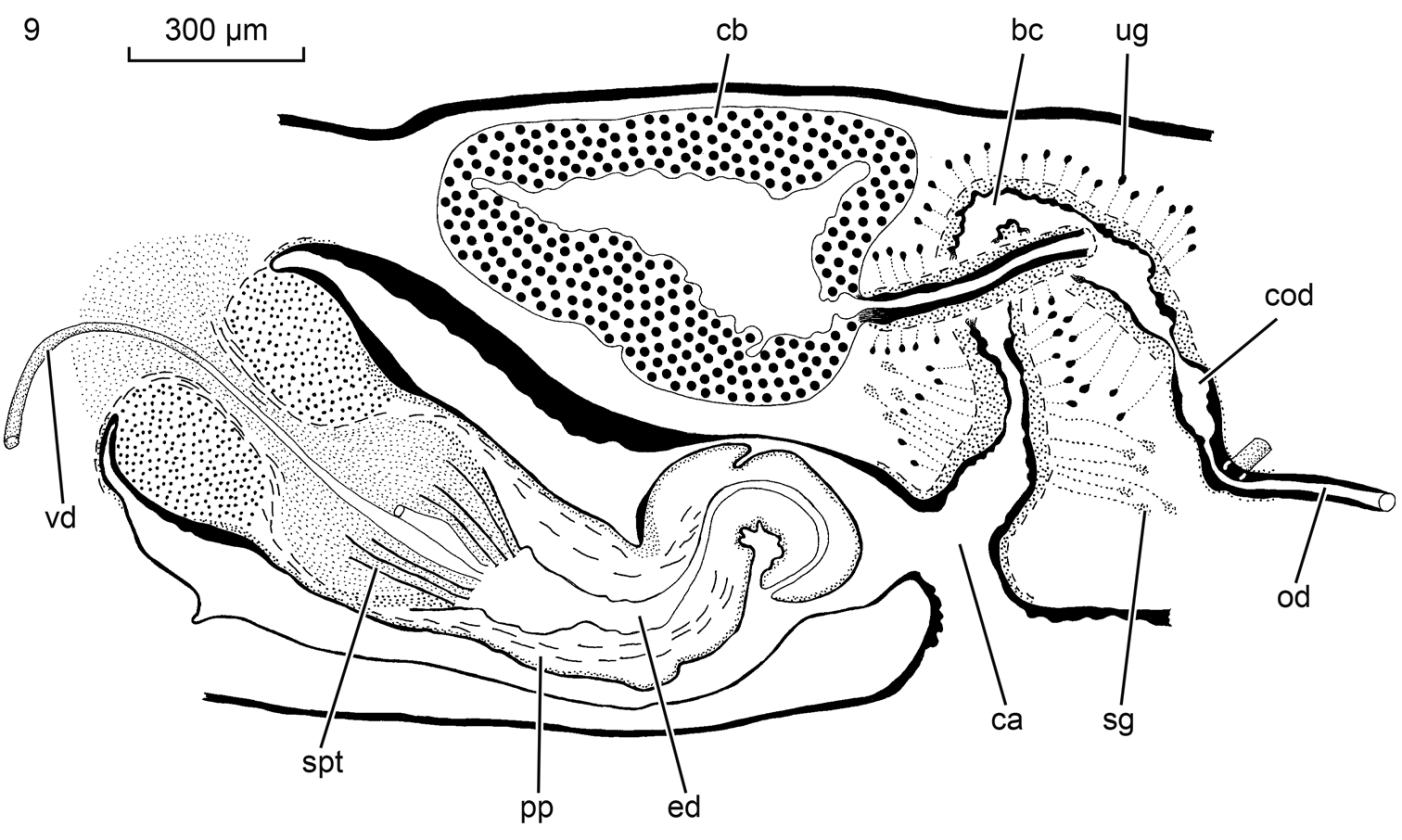
*Obrimoposthiawandeli*. NIBRIV0000813547, sagittal reconstruction of the copulatory apparatus; anterior to the left.

**Figures 10–11.**
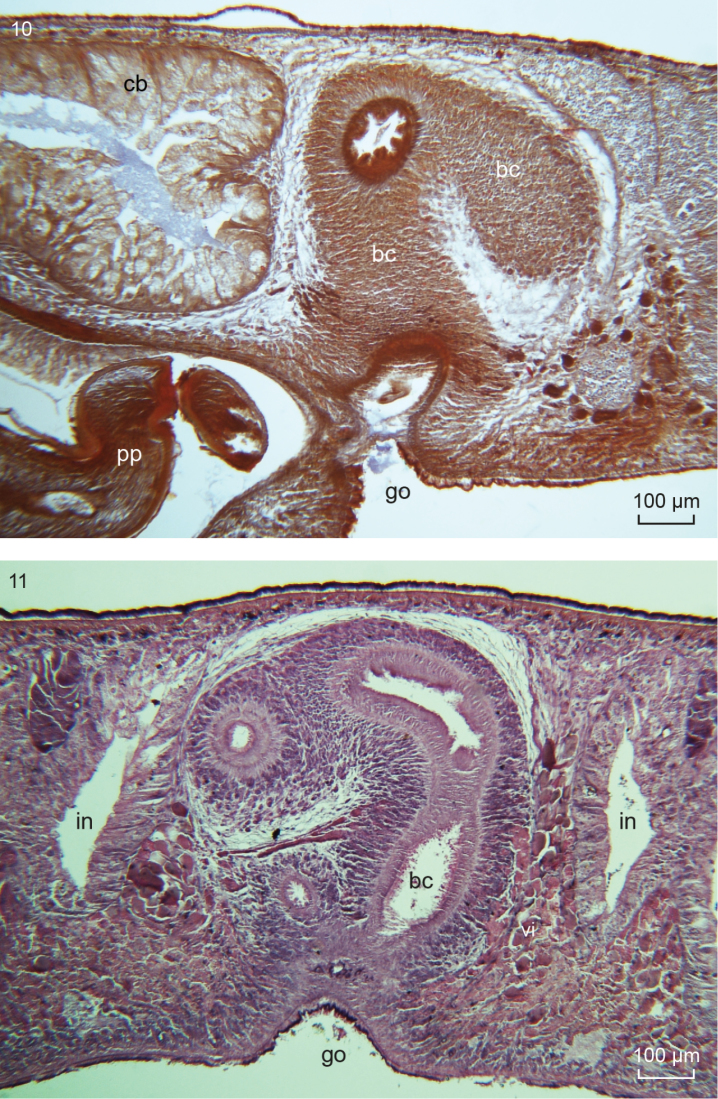
*Obrimoposthiawandeli*. **10** NIBRIV0000813547, microphotograph of sagittal section of the copulatory apparatus; anterior to the left **11**ZMA V.Pl. 951.5, microphotograph of transverse section through the bursal canal and gonopore.

**Figures 12–13.**
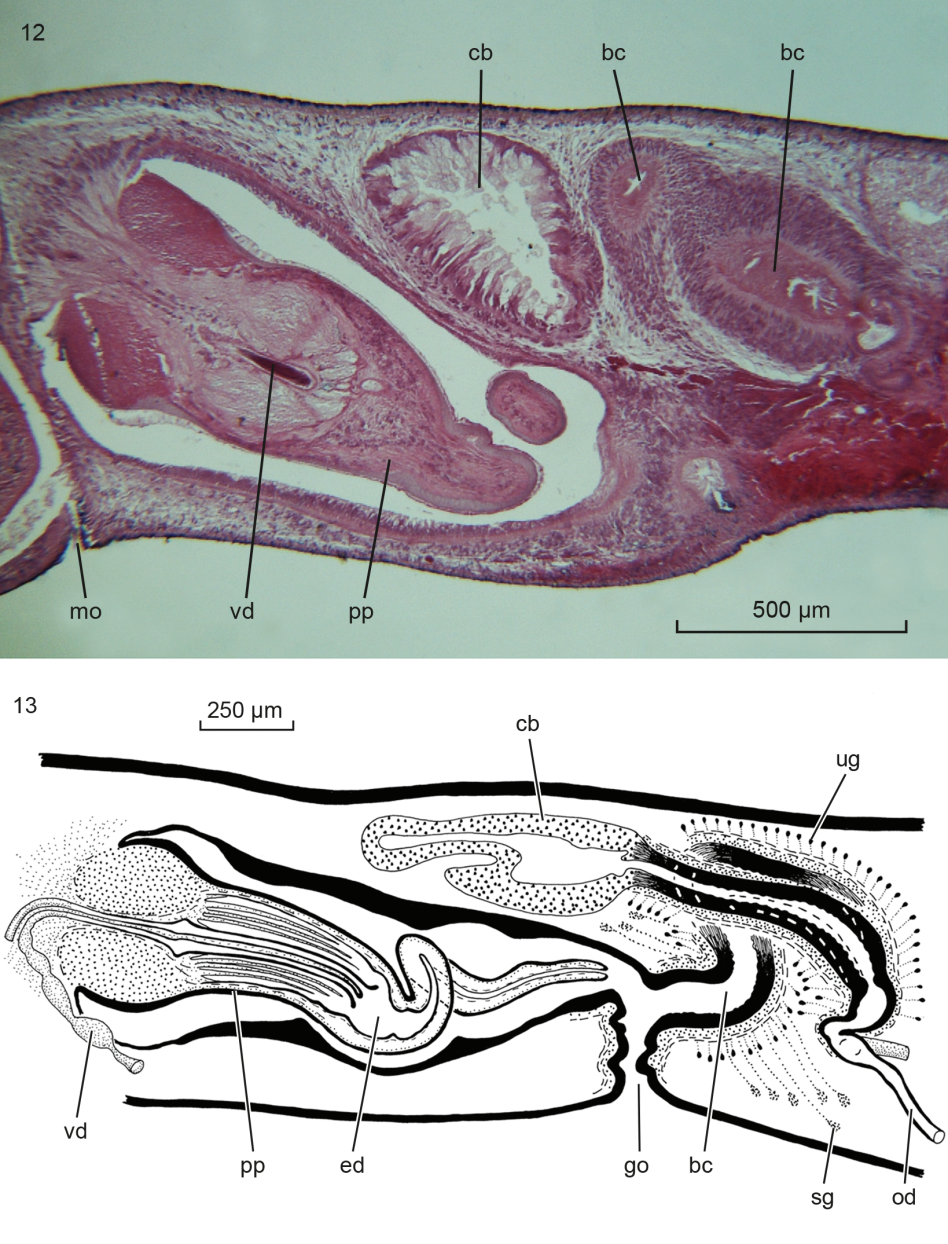
*Obrimoposthiawandeli*. **12** MZUPL 00290-A163, microphotograph of sagittal section of copulatory apparatus of holotype of *Procerodessanderi*; anterior to the left **13** MZU PL. 00291(nos. A788-821), sagittal reconstruction of the copulatory apparatus of presumed specimen of *Procerodessanderi*.

The several specimens available from the population of King George Island revealed the presence of intraspecific variability in the female reproductive system. Generally, *O.wandeli* has been described as having a bursal canal that shows a distinct T-junction, with the posterior branch of the T forming a kind of diverticulum that receives the opening of the common oviduct (see Sluys 1989, fig. 141). In some animals from King George Island such a T-junction is indeed present (ZMA V.Pl. 951.1, V.Pl. 951.4, V.Pl. 951.6). However, in others, the situation is different in that in these animals the common oviduct opens, via a constriction, into the bursal canal, which from there on runs antero-dorsad and then gently curves rather abruptly ventrad to open into the common atrium (Figs 9, 10). This portion of the bursal canal shows a clear, lateral bend during its course towards the common atrium (Figure 11), as reported earlier for *O.wandeli* (Sluys 1989). From the side of this obliquely, antero-dorsally running part of the bursal canal arises a branch that runs more or less straight forward to communicate with the copulatory bursa (Figure 9). Precisely the same situation is present in the holotype of *P.sanderi* (Figure 12). In some presumed specimens of *P.sanderi* the branch that runs to the bursa may even originate very close to the point where the common oviduct communicates with the bursal canal (Figure 13).

The entire bursal canal, including its side branch, is lined with an infranucleated epithelium and is surrounded by a thick, subepithelial layer of circular muscle, bounded by a much thinner layer of longitudinal muscle. Oviducts and common oviduct are lined with a nucleated epithelium and are surrounded by a thin layer of circular muscles. The entire bursal canal is surrounded by a broad zone of unicellular glands, which discharge their erythrophilic secretion into the canal. Erythrophilic shell glands discharge their secretion into the ventral portion of the bursal canal, near its communication with the common atrium.

##### Discussion.

In an anonymous article in a bulletin, the late Josef Hauser described the presumed new species *Procerodessanderi* [Hauser, 1987] (Anonymous 1987). That Hauser was indeed the author of this article was apparent, for example, from the fact that in 1988 and 1989 he corresponded on this subject with both Masaharu Kawakatsu and Ronald Sluys and that he forwarded to these workers photocopies of the article. Furthermore, in the article the new species is attributed to Hauser. In his article Hauser claimed that the anatomy of *P.sanderi* was different from congeneric species, including species currently assigned to the genus *Obrimoposthia*. Unfortunately, the article did not provide a reconstruction drawing of the copulatory apparatus, while the short description of the reproductive apparatus in the Portuguese language neither did make clear the anatomical differences between the new species and its congeners. Furthermore, the material that Hauser made available to both Sluys and Kawakatsu, consisting of printed photographs, histological slides, and reconstruction drawings, at the time did not convince these two workers that indeed the specimens represented a new species. As a result, in his monograph Sluys (1989) synonymized *Procerodessanderi* with *Obrimoposthiawandeli*. In their joint publication, Sluys and Kawakatsu (2005) reiterated their conclusion, as expressed also in correspondence with Hauser, that the species *P.sanderi* is synonymous with *O.wandeli*.

Nevertheless, examination of our new material collected in 2017, as well as re-examination of specimens from King George Island that were collected in 1983 and were part of Hauser’s samples, including a specimen that he had designated as the holotype specimen of *P.sanderi*, revealed that at least within this population there is clear intraspecific variation in the construction of the female copulatory apparatus.

In earlier studies (e.g., Sluys and Kawakatsu 2005) the deviant course of the bursal canal in some specimens of *O.wandeli* from King George Island, i.e. absence of the T-junction and origination of a duct from the side of the bursal canal, was not clearly observed as no reconstruction drawings were made of the various specimens. The present series of material that is available undeniably shows that the intraspecific variability of this population is exhibited by animals collected both in 1983 and 2017. Therefore, we do here consider this variability in the course of the bursal canal, as described above, to be a constant, stable feature of at least the population from King George Island and probably for other populations of *O.wandeli* as well.

One might contemplate an alternative explanation for the deviant course of the bursal canal. As the specimens from King George Island were somewhat larger than generally reported for *O.wandeli* (see above), one may view their copulatory apparatus as having reached the final stage of maturation. However, although we can envision structures becoming larger during maturation, we believe it to be unlikely for anatomical organs to become structurally different. In other words, we consider it unlikely that upon maturation a T-junction in the bursal canal will re-assemble in such a way that it develops into a duct with a distinct loop from which originates a side-branch that runs to the copulatory bursa. Therefore, we consider these different expressions of the course of the bursal canal and its connection with the copulatory bursa to be the result of intraspecific variation, independent of the stage of maturation.

In our phylogenetic tree (Figure 1) *O.wandeli* is the sister-group of the genus *Procerodes* Girard, 1850. This reflects the taxonomic history of the current members of the genus *Obrimoposthia*, most of which were formerly assigned to the genus *Procerodes*. However, it became increasingly clear that in the past the genus *Procerodes* constituted an unnatural assemblage of species that belonged to different natural groups (Sluys 1986), one such group being formed by the present members of the genus *Obrimoposthia* (Sluys and Ball 1988, Sluys 1989). This has resulted in the situation that in the most recent taxonomy of the Maricola the genera *Procerodes* and *Obrimoposthia* are even classified in different families, viz. Procerodidae Diesing, 1862 and Uteriporidae Böhmig, 1906, respectively (Sluys 1989, Sluys et al. 2009). In the phylogenetic tree of the Uteriporidae based on morphological characters, the genus *Obrimoposthia* is closely related to *Paucumara* Sluys, 1989 and *Ectoplana* Kaburaki, 1917 (Sluys 1989, fig. 302), both belonging to the subfamily Ectoplaninae Bresslau, 1933. It is clear that in our present tree (Figure 1) *Obrimoposthia* is rather far removed from *Ectoplana* and *Paucumara*.

## Supplementary Material

XML Treatment for Paucumara trigonocephala

XML Treatment for Obrimoposthia wandeli
